# 2,2-Dimethyl-5-[(5-methyl­furan-2-yl)methyl­idene]-1,3-dioxane-4,6-dione

**DOI:** 10.1107/S1600536811002285

**Published:** 2011-01-22

**Authors:** Wu-Lan Zeng

**Affiliations:** a MicroScale Science Institute, Department of Chemistry and Chemical Engineering, Weifang University, Weifang 261061, People’s Republic of China

## Abstract

The asymmetric unit of the title compound, C_12_H_12_O_5_, contains two independent mol­ecules. In each, the 1,3-dioxane ring adopts an envelope conformation with the dimethyl-substituted C atom forming the flap. The crystal structure is stabilized by weak inter­molecular C—H⋯O hydrogen bonds.

## Related literature

For related structures, see: Zeng (2010*a* ▶,*b* ▶).
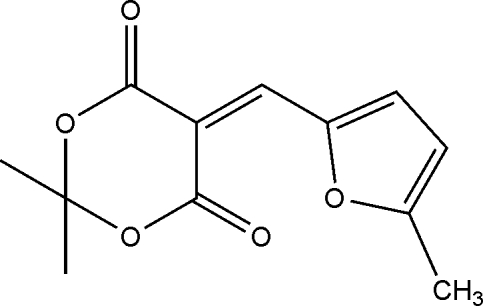


## Experimental

### 

#### Crystal data

C_12_H_12_O_5_
*M* *_r_* = 236.22
Triclinic, 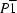

*a* = 8.9590 (18) Å
*b* = 10.038 (2) Å
*c* = 13.616 (3) Å
α = 92.71 (3)°
β = 105.99 (3)°
γ = 91.67 (3)°
*V* = 1174.7 (4) Å^3^
*Z* = 4
Mo *K*α radiation
μ = 0.11 mm^−1^
*T* = 293 K
0.20 × 0.16 × 0.12 mm

#### Data collection

Bruker SMART CCD area-detector diffractometer
11592 measured reflections
5336 independent reflections
3333 reflections with *I* > 2σ(*I*)
*R*_int_ = 0.024

#### Refinement

*R*[*F* ^2^ > 2σ(*F* ^2^)] = 0.044
*wR*(*F* ^2^) = 0.156
*S* = 1.07
5336 reflections
307 parameters
H-atom parameters constrained
Δρ_max_ = 0.31 e Å^−3^
Δρ_min_ = −0.30 e Å^−3^

### 

Data collection: *SMART* (Bruker, 1997 ▶); cell refinement: *SAINT* (Bruker, 1997 ▶); data reduction: *SAINT*; program(s) used to solve structure: *SHELXS97* (Sheldrick, 2008 ▶); program(s) used to refine structure: *SHELXL97* (Sheldrick, 2008 ▶); molecular graphics: *SHELXTL* (Sheldrick, 2008 ▶); software used to prepare material for publication: *SHELXTL*.

## Supplementary Material

Crystal structure: contains datablocks global, I. DOI: 10.1107/S1600536811002285/lh5196sup1.cif

Structure factors: contains datablocks I. DOI: 10.1107/S1600536811002285/lh5196Isup2.hkl

Additional supplementary materials:  crystallographic information; 3D view; checkCIF report

## Figures and Tables

**Table 1:** Hydrogen-bond geometry (Å, °)

*D*—H⋯*A*	*D*—H	H⋯*A*	*D*⋯*A*	*D*—H⋯*A*
C1*B*—H1*BC*⋯O3*B*^i^	0.96	2.48	3.434 (3)	170
C10*B*—H10*A*⋯O4*A*^ii^	0.93	2.60	3.448 (3)	153
C10*A*—H10*B*⋯O1*A*^iii^	0.93	2.44	3.343 (2)	163
C2*A*—H2*AB*⋯O2*A*^iv^	0.96	2.57	3.524 (3)	170
C1*A*—H1*AA*⋯O3*B*	0.96	2.55	3.496 (3)	170

Symmetry codes: (i) 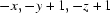; (ii) 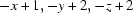; (iii) 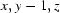; (iv) 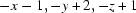.

**Fig. 1.**
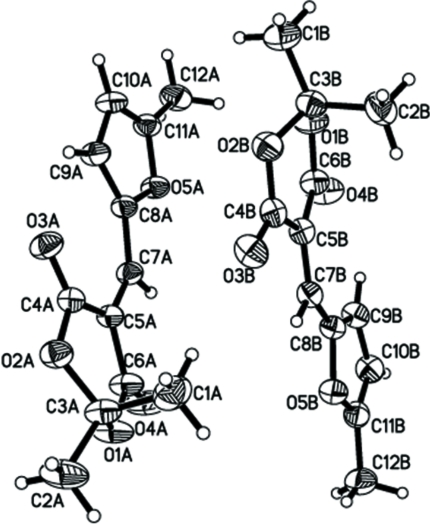
The molecular structure of (I), drawn with 30% probability ellipsoids and spheres of arbritrary size for the H atoms.

## supplementary crystallographic information

### Comment

In previous papers, the author recently reported the crystal structure of 5-(4-fluorobenzylidene)-2,2-dimethyl-1,3-dioxane-4,6-dione and (E)-2,2-dimethyl-5-(3-phenylallylidene)-1,3-dioxane-4,6- dione (Zeng, 2010*a*,*b*). As part of this search for new Meldrum's acid compounds, the title compound, (I) (Fig. 1), was synthesized and its crystal structure is reported herein. There are two symmetry-independent molecules, A and B, in the asymmetric unit of (I). The corresponding bond lengths and angles for the indepenent molecules agree well with each other as is reflected especially for the C7═C5 distance where the value and the standard uncertainty are the same in each. The 1,3-dioxane rings in each molecule are in envelope conformations. The crystal structure is stabilized by weak intermolecular C—H···O hydrogen bonds (Table 1).

### Experimental

The mixture of malonic acid (6.24 g, 0.06 mol) and acetic anhydride(9 ml) in conc. sulfuric acid (0.25 ml) was stirred with water at 303K, After dissolving, propan-2-one (3.48 g, 0.06 mol) was added dropwise into solution for 1 h. The reaction was allowed to proceed for 2 h. The mixture was cooled and filtered, and then an ethanol solution of 5-methylfuran-2-carbaldehyde (6.60g,0.06 mol) was added. The solution was then filtered and concentrated. Single crystals were obtained by evaporation of an petroleum ether-acetone (3:1 *v*/*v*) solution of (I) at room temperature over a period of several days.

### Refinement

The H atoms were placed in calculated positions (C—H = 0.93–0.96 Å), and refined as riding with U_iso_(H) = 1.2U_eq_(C) or 1.5U_eq_(methyl C).

### Crystal data

**Table:**

C_12_H_12_O_5_	*Z* = 4
*M**_r_* = 236.22	*F*(000) = 496
Triclinic, *P*1	*D*_x_ = 1.336 Mg m^−^^3^
Hall symbol: -P 1	Mo *K*α radiation, λ = 0.71073 Å
*a* = 8.9590 (18) Å	Cell parameters from 3333 reflections
*b* = 10.038 (2) Å	θ = 3.1–27.5°
*c* = 13.616 (3) Å	µ = 0.11 mm^−^^1^
α = 92.71 (3)°	*T* = 293 K
β = 105.99 (3)°	Block, yellow
γ = 91.67 (3)°	0.20 × 0.16 × 0.12 mm
*V* = 1174.7 (4) Å^3^	

### Data collection

**Table:**

Bruker SMART CCD area-detector diffractometer	3333 reflections with *I* > 2σ(*I*)
Radiation source: fine-focus sealed tube	*R*_int_ = 0.024
graphite	θ_max_ = 27.5°, θ_min_ = 3.1°
φ and ω scans	*h* = −11→10
11592 measured reflections	*k* = −13→13
5336 independent reflections	*l* = −17→17

### Refinement

**Table:**

Refinement on *F*^2^	Primary atom site location: structure-invariant direct methods
Least-squares matrix: full	Secondary atom site location: difference Fourier map
*R*[*F*^2^ > 2σ(*F*^2^)] = 0.044	Hydrogen site location: inferred from neighbouring sites
*wR*(*F*^2^) = 0.156	H-atom parameters constrained
*S* = 1.07	*w* = 1/[σ^2^(*F*_o_^2^) + (0.0907*P*)^2^] where *P* = (*F*_o_^2^ + 2*F*_c_^2^)/3
5336 reflections	(Δ/σ)_max_ = 0.001
307 parameters	Δρ_max_ = 0.31 e Å^−^^3^
0 restraints	Δρ_min_ = −0.30 e Å^−^^3^

### Special details

**Table:**

Geometry. All e.s.d.'s (except the e.s.d. in the dihedral angle between two l.s. planes) are estimated using the full covariance matrix. The cell e.s.d.'s are taken into account individually in the estimation of e.s.d.'s in distances, angles and torsion angles; correlations between e.s.d.'s in cell parameters are only used when they are defined by crystal symmetry. An approximate (isotropic) treatment of cell e.s.d.'s is used for estimating e.s.d.'s involving l.s. planes.
Refinement. Refinement of *F*^2^ against ALL reflections. The weighted *R*-factor *wR* and goodness of fit *S* are based on *F*^2^, conventional *R*-factors *R* are based on *F*, with *F* set to zero for negative *F*^2^. The threshold expression of *F*^2^ > σ(*F*^2^) is used only for calculating *R*-factors(gt) *etc*. and is not relevant to the choice of reflections for refinement. *R*-factors based on *F*^2^ are statistically about twice as large as those based on *F*, and *R*- factors based on ALL data will be even larger.

### Fractional atomic coordinates and isotropic or equivalent isotropic displacement parameters (Å^2^)

**Table:**

	*x*	*y*	*z*	*U*_iso_*/*U*_eq_	
O2B	0.11969 (13)	0.55646 (11)	0.62210 (9)	0.0618 (3)	
O5B	0.45369 (14)	1.05664 (10)	0.82774 (9)	0.0563 (3)	
O1B	0.34137 (15)	0.48325 (11)	0.74003 (9)	0.0645 (3)	
C5B	0.29935 (18)	0.71777 (15)	0.73236 (11)	0.0467 (4)	
O3B	0.06239 (14)	0.76729 (12)	0.61279 (9)	0.0671 (3)	
O4B	0.48827 (17)	0.61623 (12)	0.86197 (9)	0.0749 (4)	
C6B	0.3859 (2)	0.60755 (16)	0.78319 (12)	0.0536 (4)	
C7B	0.33715 (18)	0.84921 (15)	0.75839 (11)	0.0485 (4)	
H7BA	0.2651	0.9054	0.7206	0.058*	
C4B	0.15441 (19)	0.68642 (16)	0.65155 (12)	0.0522 (4)	
C8B	0.46201 (19)	0.91860 (14)	0.83032 (11)	0.0481 (4)	
C9B	0.5954 (2)	0.89135 (17)	0.90245 (12)	0.0564 (4)	
H9BA	0.6302	0.8070	0.9202	0.068*	
C3B	0.2442 (2)	0.46641 (16)	0.63735 (13)	0.0573 (4)	
C11B	0.5813 (2)	1.11096 (17)	0.89765 (13)	0.0569 (4)	
C10B	0.6699 (2)	1.01280 (18)	0.94451 (14)	0.0622 (4)	
H10A	0.7635	1.0245	0.9955	0.075*	
C1B	0.1707 (3)	0.32824 (18)	0.62731 (17)	0.0801 (6)	
H1BA	0.1141	0.3201	0.6774	0.120*	
H1BB	0.2501	0.2640	0.6381	0.120*	
H1BC	0.1008	0.3123	0.5600	0.120*	
C2B	0.3372 (3)	0.4908 (2)	0.56325 (16)	0.0780 (6)	
H2BA	0.3813	0.5805	0.5749	0.117*	
H2BB	0.2708	0.4787	0.4946	0.117*	
H2BC	0.4191	0.4290	0.5728	0.117*	
C12B	0.5961 (3)	1.25844 (18)	0.90908 (17)	0.0785 (6)	
H12A	0.5088	1.2952	0.8619	0.118*	
H12B	0.6903	1.2885	0.8950	0.118*	
H12C	0.5986	1.2874	0.9778	0.118*	
O5A	0.07339 (13)	0.59957 (9)	0.88357 (8)	0.0527 (3)	
O2A	−0.36509 (13)	0.95250 (10)	0.63621 (9)	0.0573 (3)	
O3A	−0.35857 (14)	0.73997 (11)	0.66614 (9)	0.0617 (3)	
C5A	−0.14448 (18)	0.87879 (13)	0.76199 (11)	0.0447 (3)	
O1A	−0.17387 (16)	1.11015 (10)	0.72684 (10)	0.0698 (4)	
C8A	−0.05825 (18)	0.64850 (14)	0.81836 (11)	0.0447 (3)	
C7A	−0.05103 (18)	0.78842 (14)	0.81679 (12)	0.0468 (4)	
H7AA	0.0371	0.8277	0.8635	0.056*	
C4A	−0.29180 (18)	0.84726 (14)	0.68578 (11)	0.0461 (4)	
C6A	−0.0910 (2)	1.01900 (15)	0.78701 (14)	0.0596 (4)	
O4A	0.01460 (18)	1.05984 (12)	0.85763 (12)	0.0924 (5)	
C11A	0.0546 (2)	0.46486 (14)	0.87915 (13)	0.0529 (4)	
C9A	−0.1573 (2)	0.54271 (14)	0.77519 (13)	0.0556 (4)	
H9AA	−0.2548	0.5464	0.7287	0.067*	
C3A	−0.2727 (2)	1.06729 (15)	0.62802 (13)	0.0592 (4)	
C10A	−0.0843 (2)	0.42757 (15)	0.81423 (14)	0.0614 (5)	
H10B	−0.1249	0.3404	0.7981	0.074*	
C2A	−0.3843 (3)	1.17833 (18)	0.59790 (17)	0.0892 (7)	
H2AA	−0.4385	1.1928	0.6490	0.134*	
H2AB	−0.4578	1.1538	0.5332	0.134*	
H2AC	−0.3273	1.2588	0.5924	0.134*	
C12A	0.1835 (2)	0.39080 (17)	0.94262 (15)	0.0713 (5)	
H12D	0.1569	0.2968	0.9333	0.107*	
H12E	0.2009	0.4190	1.0134	0.107*	
H12F	0.2763	0.4085	0.9223	0.107*	
C1A	−0.1792 (3)	1.0368 (2)	0.55522 (18)	0.0901 (7)	
H1AA	−0.1115	0.9659	0.5798	0.135*	
H1AB	−0.1182	1.1149	0.5499	0.135*	
H1AC	−0.2475	1.0099	0.4891	0.135*	

### Atomic displacement parameters (Å^2^)

**Table:**

	*U*^11^	*U*^22^	*U*^33^	*U*^12^	*U*^13^	*U*^23^
O2B	0.0499 (7)	0.0631 (7)	0.0632 (7)	−0.0012 (5)	0.0031 (6)	−0.0101 (6)
O5B	0.0605 (7)	0.0498 (6)	0.0555 (6)	0.0040 (5)	0.0110 (6)	0.0001 (5)
O1B	0.0751 (9)	0.0494 (6)	0.0566 (7)	0.0003 (5)	−0.0020 (6)	0.0017 (5)
C5B	0.0457 (9)	0.0530 (8)	0.0388 (8)	0.0032 (6)	0.0071 (7)	0.0021 (6)
O3B	0.0566 (8)	0.0765 (8)	0.0591 (7)	0.0159 (6)	0.0000 (6)	0.0020 (6)
O4B	0.0855 (10)	0.0592 (7)	0.0587 (8)	0.0038 (6)	−0.0165 (7)	0.0077 (6)
C6B	0.0573 (10)	0.0513 (9)	0.0462 (9)	0.0001 (7)	0.0048 (8)	0.0017 (7)
C7B	0.0483 (9)	0.0521 (8)	0.0441 (8)	0.0068 (7)	0.0102 (7)	0.0045 (7)
C4B	0.0492 (9)	0.0614 (9)	0.0452 (8)	0.0050 (7)	0.0119 (7)	0.0006 (7)
C8B	0.0540 (9)	0.0444 (8)	0.0448 (8)	0.0049 (6)	0.0119 (7)	0.0017 (6)
C9B	0.0564 (10)	0.0574 (9)	0.0510 (9)	0.0078 (7)	0.0073 (8)	0.0010 (8)
C3B	0.0585 (11)	0.0549 (9)	0.0519 (9)	0.0040 (7)	0.0055 (8)	−0.0035 (7)
C11B	0.0587 (11)	0.0580 (10)	0.0537 (9)	−0.0044 (8)	0.0174 (8)	−0.0079 (8)
C10B	0.0556 (11)	0.0672 (11)	0.0557 (10)	−0.0018 (8)	0.0041 (8)	−0.0068 (8)
C1B	0.0866 (15)	0.0621 (11)	0.0799 (13)	−0.0109 (10)	0.0070 (12)	−0.0092 (10)
C2B	0.0880 (15)	0.0808 (13)	0.0711 (12)	0.0114 (11)	0.0326 (12)	−0.0039 (10)
C12B	0.0895 (16)	0.0570 (11)	0.0872 (14)	−0.0067 (10)	0.0246 (12)	−0.0075 (10)
O5A	0.0561 (7)	0.0374 (5)	0.0592 (7)	0.0039 (4)	0.0061 (5)	0.0076 (5)
O2A	0.0550 (7)	0.0492 (6)	0.0600 (7)	0.0048 (5)	0.0018 (5)	0.0103 (5)
O3A	0.0549 (7)	0.0477 (6)	0.0712 (8)	−0.0089 (5)	0.0001 (6)	0.0026 (5)
C5A	0.0480 (9)	0.0354 (7)	0.0479 (8)	0.0017 (6)	0.0090 (7)	0.0018 (6)
O1A	0.0849 (9)	0.0341 (5)	0.0708 (8)	0.0066 (5)	−0.0116 (7)	0.0015 (5)
C8A	0.0480 (9)	0.0367 (7)	0.0473 (8)	0.0040 (6)	0.0092 (7)	0.0059 (6)
C7A	0.0463 (9)	0.0392 (7)	0.0506 (8)	−0.0023 (6)	0.0073 (7)	−0.0013 (6)
C4A	0.0493 (9)	0.0410 (8)	0.0469 (8)	0.0027 (6)	0.0113 (7)	0.0024 (6)
C6A	0.0624 (11)	0.0376 (8)	0.0660 (11)	0.0023 (7)	−0.0038 (9)	0.0035 (7)
O4A	0.0940 (11)	0.0423 (6)	0.1015 (11)	−0.0076 (6)	−0.0370 (9)	0.0016 (7)
C11A	0.0673 (11)	0.0376 (7)	0.0568 (9)	0.0069 (7)	0.0209 (9)	0.0080 (7)
C9A	0.0588 (10)	0.0407 (8)	0.0623 (10)	−0.0016 (7)	0.0094 (8)	0.0012 (7)
C3A	0.0724 (12)	0.0395 (8)	0.0562 (10)	0.0010 (7)	0.0018 (9)	0.0059 (7)
C10A	0.0763 (13)	0.0325 (7)	0.0738 (11)	−0.0008 (7)	0.0185 (10)	0.0026 (7)
C2A	0.1048 (18)	0.0537 (11)	0.0846 (14)	0.0204 (10)	−0.0177 (12)	0.0118 (10)
C12A	0.0846 (14)	0.0530 (10)	0.0762 (12)	0.0200 (9)	0.0180 (11)	0.0194 (9)
C1A	0.1165 (19)	0.0727 (13)	0.0917 (15)	−0.0048 (12)	0.0447 (15)	0.0214 (12)

### Geometric parameters (Å, °)

**Table:**

O2B—C4B	1.348 (2)	O5A—C11A	1.3542 (17)
O2B—C3B	1.433 (2)	O5A—C8A	1.3851 (18)
O5B—C11B	1.351 (2)	O2A—C4A	1.3662 (18)
O5B—C8B	1.3914 (17)	O2A—C3A	1.425 (2)
O1B—C6B	1.354 (2)	O3A—C4A	1.1985 (19)
O1B—C3B	1.428 (2)	C5A—C7A	1.358 (2)
C5B—C7B	1.358 (2)	C5A—C4A	1.452 (2)
C5B—C6B	1.460 (2)	C5A—C6A	1.467 (2)
C5B—C4B	1.467 (2)	O1A—C6A	1.3545 (19)
O3B—C4B	1.2070 (19)	O1A—C3A	1.431 (2)
O4B—C6B	1.202 (2)	C8A—C9A	1.364 (2)
C7B—C8B	1.408 (2)	C8A—C7A	1.4056 (19)
C7B—H7BA	0.9300	C7A—H7AA	0.9300
C8B—C9B	1.366 (2)	C6A—O4A	1.194 (2)
C9B—C10B	1.391 (2)	C11A—C10A	1.344 (3)
C9B—H9BA	0.9300	C11A—C12A	1.481 (2)
C3B—C2B	1.499 (3)	C9A—C10A	1.399 (2)
C3B—C1B	1.502 (2)	C9A—H9AA	0.9300
C11B—C10B	1.354 (2)	C3A—C1A	1.491 (3)
C11B—C12B	1.480 (2)	C3A—C2A	1.514 (2)
C10B—H10A	0.9300	C10A—H10B	0.9300
C1B—H1BA	0.9600	C2A—H2AA	0.9600
C1B—H1BB	0.9600	C2A—H2AB	0.9600
C1B—H1BC	0.9600	C2A—H2AC	0.9600
C2B—H2BA	0.9600	C12A—H12D	0.9600
C2B—H2BB	0.9600	C12A—H12E	0.9600
C2B—H2BC	0.9600	C12A—H12F	0.9600
C12B—H12A	0.9600	C1A—H1AA	0.9600
C12B—H12B	0.9600	C1A—H1AB	0.9600
C12B—H12C	0.9600	C1A—H1AC	0.9600
			
C4B—O2B—C3B	118.43 (13)	C11A—O5A—C8A	107.77 (13)
C11B—O5B—C8B	107.37 (13)	C4A—O2A—C3A	118.26 (13)
C6B—O1B—C3B	119.91 (13)	C7A—C5A—C4A	125.38 (14)
C7B—C5B—C6B	124.95 (15)	C7A—C5A—C6A	115.42 (15)
C7B—C5B—C4B	116.48 (14)	C4A—C5A—C6A	119.16 (13)
C6B—C5B—C4B	118.43 (15)	C6A—O1A—C3A	119.26 (12)
O4B—C6B—O1B	116.99 (15)	C9A—C8A—O5A	108.02 (13)
O4B—C6B—C5B	126.11 (16)	C9A—C8A—C7A	139.38 (15)
O1B—C6B—C5B	116.81 (14)	O5A—C8A—C7A	112.60 (13)
C5B—C7B—C8B	133.72 (14)	C5A—C7A—C8A	134.05 (16)
C5B—C7B—H7BA	113.1	C5A—C7A—H7AA	113.0
C8B—C7B—H7BA	113.1	C8A—C7A—H7AA	113.0
O3B—C4B—O2B	118.18 (16)	O3A—C4A—O2A	117.16 (15)
O3B—C4B—C5B	124.96 (16)	O3A—C4A—C5A	126.53 (14)
O2B—C4B—C5B	116.81 (14)	O2A—C4A—C5A	116.19 (13)
C9B—C8B—O5B	107.91 (15)	O4A—C6A—O1A	117.49 (15)
C9B—C8B—C7B	138.86 (15)	O4A—C6A—C5A	125.89 (14)
O5B—C8B—C7B	113.20 (13)	O1A—C6A—C5A	116.55 (15)
C8B—C9B—C10B	107.48 (16)	C10A—C11A—O5A	109.20 (14)
C8B—C9B—H9BA	126.3	C10A—C11A—C12A	133.71 (15)
C10B—C9B—H9BA	126.3	O5A—C11A—C12A	117.09 (16)
O1B—C3B—O2B	110.13 (13)	C8A—C9A—C10A	106.92 (16)
O1B—C3B—C2B	110.27 (16)	C8A—C9A—H9AA	126.5
O2B—C3B—C2B	109.85 (15)	C10A—C9A—H9AA	126.5
O1B—C3B—C1B	105.73 (14)	O2A—C3A—O1A	109.72 (13)
O2B—C3B—C1B	106.35 (16)	O2A—C3A—C1A	110.21 (15)
C2B—C3B—C1B	114.36 (16)	O1A—C3A—C1A	110.72 (17)
O5B—C11B—C10B	109.69 (15)	O2A—C3A—C2A	106.18 (16)
O5B—C11B—C12B	116.80 (16)	O1A—C3A—C2A	105.14 (14)
C10B—C11B—C12B	133.51 (18)	C1A—C3A—C2A	114.63 (17)
C11B—C10B—C9B	107.55 (17)	C11A—C10A—C9A	108.08 (15)
C11B—C10B—H10A	126.2	C11A—C10A—H10B	126.0
C9B—C10B—H10A	126.2	C9A—C10A—H10B	126.0
C3B—C1B—H1BA	109.5	C3A—C2A—H2AA	109.5
C3B—C1B—H1BB	109.5	C3A—C2A—H2AB	109.5
H1BA—C1B—H1BB	109.5	H2AA—C2A—H2AB	109.5
C3B—C1B—H1BC	109.5	C3A—C2A—H2AC	109.5
H1BA—C1B—H1BC	109.5	H2AA—C2A—H2AC	109.5
H1BB—C1B—H1BC	109.5	H2AB—C2A—H2AC	109.5
C3B—C2B—H2BA	109.5	C11A—C12A—H12D	109.5
C3B—C2B—H2BB	109.5	C11A—C12A—H12E	109.5
H2BA—C2B—H2BB	109.5	H12D—C12A—H12E	109.5
C3B—C2B—H2BC	109.5	C11A—C12A—H12F	109.5
H2BA—C2B—H2BC	109.5	H12D—C12A—H12F	109.5
H2BB—C2B—H2BC	109.5	H12E—C12A—H12F	109.5
C11B—C12B—H12A	109.5	C3A—C1A—H1AA	109.5
C11B—C12B—H12B	109.5	C3A—C1A—H1AB	109.5
H12A—C12B—H12B	109.5	H1AA—C1A—H1AB	109.5
C11B—C12B—H12C	109.5	C3A—C1A—H1AC	109.5
H12A—C12B—H12C	109.5	H1AA—C1A—H1AC	109.5
H12B—C12B—H12C	109.5	H1AB—C1A—H1AC	109.5

### Hydrogen-bond geometry (Å, °)

**Table:**

*D*—H···*A*	*D*—H	H···*A*	*D*···*A*	*D*—H···*A*
C1B—H1BC···O3B^i^	0.96	2.48	3.434 (3)	170
C10B—H10A···O4A^ii^	0.93	2.60	3.448 (3)	153
C10A—H10B···O1A^iii^	0.93	2.44	3.343 (2)	163
C2A—H2AB···O2A^iv^	0.96	2.57	3.524 (3)	170
C1A—H1AA···O3B	0.96	2.55	3.496 (3)	170

Symmetry codes: (i) −*x*, −*y*+1, −*z*+1; (ii) −*x*+1, −*y*+2, −*z*+2; (iii) *x*, *y*−1, *z*; (iv) −*x*−1, −*y*+2, −*z*+1.

## References

[bb1] Bruker (1997). *SMART* and *SAINT* Bruker AXS Inc., Madison, Wisconsin, USA.

[bb2] Sheldrick, G. M. (2008). *Acta Cryst.* A**64**, 112–122. 10.1107/S0108767307043930 18156677

[bb3] Zeng, W.-L. (2010*a*). *Acta Cryst.* E**66**, o2366. 10.1107/S1600536810033155 PMC3008134 21588707

[bb4] Zeng, W.-L. (2010*b*). *Acta Cryst.* E**66**, o2943. 10.1107/S1600536810042534 PMC3009221 21589113

